# Characterization of the Intestinal Lactobacilli Community following Galactooligosaccharides and Polydextrose Supplementation in the Neonatal Piglet

**DOI:** 10.1371/journal.pone.0135494

**Published:** 2015-08-14

**Authors:** Jennifer L. Hoeflinger, Dimitri O. Kashtanov, Stephen B. Cox, Scot E. Dowd, Zeina E. Jouni, Sharon M. Donovan, Michael J. Miller

**Affiliations:** 1 Department of Food Science and Human Nutrition Sciences, University of Illinois Urbana Champaign, 905 S. Goodwin Ave., Urbana, IL, 61801, United States of America; 2 Division of Nutritional Sciences University of Illinois Urbana Champaign, 905 S. Goodwin Ave., Urbana, IL, 61801, United States of America; 3 Research and Testing Laboratory, 4321 Marsha Sharp FWY, Lubbock, TX, 79407, United States of America; 4 Mead Johnson Nutrition, 400 West Lloyd Expressway, Evansville, IN, 47712, United States of America; University of Louisville School of Medicine, UNITED STATES

## Abstract

Recently, prebiotic supplementation of infant formula has become common practice; however the impact on the intestinal microbiota has not been completely elucidated. In this study, neonatal piglets were randomized to: formula (FORM, n = 8), formula supplemented with 2 g/L each galactooligosaccharides (GOS) and polydextrose (PDX, F+GP, n = 9) or a sow-reared (SOW, n = 12) reference group for 19 days. The ileal (IL) and ascending colon (AC) microbiota were characterized using culture-dependent and -independent methods. 16S amplicon sequencing identified no differences at the genera level in the IL. Interestingly, six genera in the AC were significantly different between FORM and F+GP (P<0.05): *Lactobacillus*, *Ruminococcus*, *Parabacteroides*, *Oscillospira*, *Hydrogenoanaerobacterium* and *Catabacter*. In particular, the relative abundance of AC *Lactobacillus* was higher (P = 0.04) in F+GP as compared to FORM. Culture-dependent analysis of the IL and AC lactobacilli communities of FORM and F+GP revealed a *Lactobacillus* spp. composition similar to 16S amplicon sequencing. Additional analysis demonstrated individual *Lactobacillus* isolates were unable to ferment PDX. Conversely, a majority of lactobacilli isolates could ferment GOS, regardless of piglet diet. In addition, the ability of lactobacilli isolates to ferment the longer chain GOS fragments (DP 3 or greater), which are expected to be present in the distal intestine, was not different between FORM and F+GP. In conclusion, prebiotic supplementation of formula impacted the AC microbiota; however, direct utilization of GOS or PDX does not lead to an increase in *Lactobacillus* spp.

## Introduction

Infants are readily colonized following birth from exposure to the environment, most notably, microbiota from their mother’s vagina, skin, feces and potentially human milk[1–4]. After initial colonization, the infant microbiota is assembled through a complex succession of microbiota that is rapidly changing [5,6]. Additionally, the intestinal microbiota is influenced by infant nutrition [7]. Human milk is the gold standard for infant nutrition; however, formula feeding early in life is common and often required. Human milk oligosaccharides (HMOs) and their related glycoproteins and glycolipids are a significant component of human breast milk [8]. HMOs are a complex mixture of structurally diverse oligosaccharides that influence the gut microbial community, reviewed by Coppa and colleagues [9]. Bovine milk-based infant formulas are nearly devoid of complex oligosaccharides [10,11], which may negatively influence microbial colonization. Therefore, there has been interest to supplement infant formulas with alternative prebiotic oligosaccharides to provide similar functional benefits as HMOs [12–14].

Prebiotics are non-digestible carbohydrates that stimulate the growth of beneficial bacteria, thereby providing a health benefit to the host [14–17]. Several prebiotics are generally recognized as safe (GRAS) and are used as food ingredients, such as galactooligosaccharides (GOS), polydextrose (PDX) and fructooligosaccharides (FOS) [17–19]. Prebiotic supplementation of infant formula is well tolerated and stool softness and frequency are similar to breast-fed reference groups [20–22]. Additionally, GOS, commonly supplemented with FOS, increases counts of bifidobacteria [14,20,22,23] and to a lesser extent lactobacilli [23] in infant feces. A recent study reported a dose-dependent increase in lactobacilli in response to PDX supplementation in neonatal piglets [24]. Both bifidobacteria and lactobacilli have been used as commercial probiotics [25] and it is hypothesized that their health promoting functions in the gut may be enhanced by prebiotic supplementation.

Prebiotic supplemented formula is best suited for testing in infants; however, microbial community analysis is limited to feces. Therefore, a suitable analogue for infant studies is the neonatal piglet as it allows for more invasive sampling [26–28]. Findings in human infants show that bovine milk-based formula supplemented with prebiotics does not negatively affect piglet development [24,29–32]. While several studies have investigated the impact of prebiotics on the piglet gut microbiota by older traditional and molecular techniques [24,29,30,32,33], there remains a need for a more descriptive analysis.

In this study, vaginally-delivered neonatal piglets were used to model the development of the infant microbiota in response to GOS/PDX supplemented formula. We utilized culture-dependent and -independent methods to perform a broad analysis of the ileal and colonic microbiota. Additionally, we report several species of lactobacilli that are responsive to GOS supplementation.

## Materials and Methods

### Ethics statement

The experimental procedure and the use of animals were approved by the University of Illinois Institutional Animal Care and Use Committee (IACUC) in accordance with all laws and regulations pertaining to research with animals. The approved protocol was #09268.

### Animal and diets

Neonatal piglets (n = 29) were obtained from the University of Illinois Swine Research Center at 48 h-of-age to allow for ingestion of colostrum. Piglets were transported to animal facilities on campus and were individually-housed in custom cages in rooms maintained at 25°C with 12 h light-dark cycles. Additional heat was provided to an ambient temperature of 30°C within the piglet cages. The study was conducted in one replicate and used 3 litters (littermates were randomized into each treatment group). All piglets were randomized to receive a non-medicated bovine milk-based formula (Advance Baby Pig Liqui-Wean, Milk Specialties Company, Dundee, IL) alone (FORM, n = 8) or formula supplemented with 2 g/L each GOS (FrieslandCampina, Meppel, Netherlands) and PDX (Danisco, Tarrytown, NY, USA) (F+GP, n = 9). Sow-reared (SOW, n = 12) piglets were included as a reference group. Formula was offered immediately after transport to the animal facility and delivered twenty-times daily by pump for a total volume of 360 mL/kg/d for 19 d.

### Sample collection

On d 21, the piglets were lightly sedated with Telazol (7 mg/kg bodyweight; Fort Dodge Animal Health, Fort Dodge, IA, USA) and were euthanized by an intracardiac injection of sodium pentobarbital (72 mg/kg bodyweight; Fatal Plus, Vortech Pharmaceuticals, Ltd., Dearborn, MI, USA). Blood and tissue samples were collected for analysis as previously described [32]. Briefly, the small intestine was quickly excised from the pyloric sphincter to the ileocecal valve and its total length was measured. The intestine was cut into three segments at 10% and 85% of the total small intestine length to separate duodenum, jejunum and ileum. Ileal and AC contents were collected for bacterial enumeration, measurement of pH and percent dry matter or were flash frozen and stored at -80°C for 16S amplicon sequencing. The pH was measured using meter (Mettler Toledo, Columbus, OH, USA) equipped with a combination pH electrode (Hanna Instruments, Woonsocket, RI, USA). Percent dry matter of IL and AC contents was calculated following drying at 100°C for 24 h. Following the removal of contents, intestinal sections were flushed with ice-cold phosphate buffered saline (PBS) and fixed in 10% (v/v) formalin.

### Intestinal histomorphology

Formalin-fixed IL and AC tissue samples were embedded in paraffin wax, sliced to approximately 5 μm with a Leica RM2255 microtome (Leica Geosystems, Norcross, GA, USA), mounted on glass microscope slides and stained with hematoxylin and eosin. Images were captured using the Nanozoomer Digital Pathology suite (Hamamatsu, Bridgewater, NJ, USA) and measurements were determined with AxioVision (Carl Zeiss Microscopy, Thornwood, NY, USA). Images were analyzed for 8–10 well formed villi or cuffs. IL villus height and crypt depth; AC cuff width and depth were measured.

### Fermentation products

IL and AC contents were analyzed for volatile fatty acid (VFA) products by gas chromatography, as previously described [34]. VFAs measured were acetate, propionate and butyrate (short-chain fatty acids [SCFA]). Additionally, several branch-chain fatty acids (BCFA) isobutyrate, isovalerate and valerate were measured.

### Culture-dependent characterization of the microbiome

#### Bacterial enumeration

Ileal and AC contents were homogenized in PBS by vortex and aseptically plated in triplicate using an Eddy Jet spiral plater (Neu-Tec, Farmingdale, NY, USA) on *Lactobacillus* selective (LBS) agar (BD, Franklin Lakes, NJ, USA). The agar plates were incubated for 48 h at 37°C anaerobically (5% CO_2_, 5% H_2_ and 90% N_2_). Bacteria were counted using the spiral plater manufacturer’s methods and expressed as colony forming units (CFU) per gram of homogenized sample. Ten colonies per piglet per sample were randomly selected, cultivated in MRS, resuspended in 12.5% glycerol and stored at -80°C.

#### DNA Extraction

Putative lactobacilli isolates were then grown in 2 mL MRS to stationary phase. Following growth, bacterial genomic DNA was isolated using a modified bead-beating technique [35]. Briefly, 2 mL of cells were pelleted at 10,000 x g and washed twice with molecular biology grade water (Mo Bio Laboratories, Carlsbad, CA, USA). The pellets were resuspended in 750 μL of 0.5 M EDTA buffer pH 8. The resuspended cells were subjected to bead-beating in a FastPrep-24 Instrument (MP Biomedicals, Solon, OH, USA) at 6 m/s for 1 min. The samples were then centrifuged at 10,000 x g at 4°C for 3 min. A 600 μL aliquot of the supernatant was removed and frozen at -20°C for subsequent analysis.

#### Identification

A partial HSP60 sequence was amplified using lactobacilli specific HSP60 primers, LB308F (5’-TGAAGAAYGTNRYNGCYGG-3’) and LB806RM (5’-AANGTNCCVCGVATCTTGTT-3’), previously described by Blaiotta and colleagues [36]. The PCR amplification of a 499 bp amplicon was performed with a 50 μL total volume. The reaction system consisted of 3 μL of target DNA, 5 μL of each primer (10 mM), 25 μL of EconoTaq Plus Green 2X Master Mix (Lucigen Corporation, Middleton, WI, USA). The PCR consisted of an initial step of 2 min at 95°C, followed by 35 cycles (30 s at 95°C, 30 s at 50°C, and 1.5 min at 72°C) and completed with an extension step at 72°C for 15 min. PCR products were purified using the Zymoclean DNA Clean & Concentrator Kit (Zymo Research Corp., Irvine, CA, USA) according to the manufacturer's instructions. PCR products were quantified using a NanoDrop ND-1000 Spectrophotometer (NanoDrop, Wilmington, DE, USA). Sanger sequencing was done with either primer (LB308F or LB806RM) using an ABI 3730XL capillary sequencers (Life Technologies). The sequences were trimmed for quality and uploaded to the BLAST database (NCBI) to determine identity. Species identity was determined at 97% sequence similarity.

To confirm identity, a few isolates from each identified species was further sequenced by amplification of a partial 16S rRNA sequence by using the 16S rRNA primers, 8F (5’-AGAGTTTGATCCTGGCTCAG-3’) [37] and 1392R (5’-GACGGGCGGTGWGTRCA-3’) [38]. The same PCR conditions, sequencing and identification was performed as outlined above.

#### In vitro carbohydrate utilization

Lactobacilli isolated from IL and AC were tested for the ability to utilize glucose (Fisher Scientific), Purimune galactooligosaccharides (GOSP, Ingredion, Westchester, IL, USA), and PDX as the sole carbohydrate source. In preparation for the assay, isolates were revived from -80°C storage by culturing in 2 mL of MRS anaerobically for 24 or 48 h. Isolates were passaged (1%, v/v) one time into 2 mL semi-synthetic MRS medium (sMRS) containing per liter: 20 g peptone, 5 g yeast extract, 5 g sodium acetate, 2 g disodium phosphate, 2 g ammonium citrate, 1 mL tween 80, 0.1 g magnesium sulfate, 0.05 g manganese sulfate. Glucose (10 g/L) was added when necessary. Isolates were further incubated overnight (16–24 h). Following growth, a 1 mL aliquot of sMRS grown cells were washed twice using PBS, pH 7.0 by centrifugation at 13,000 x g for 5 min and resuspended in 1 mL sterile carbohydrate-free sMRS.

Stock sMRS solutions containing either a 2% (w/v) of the carbohydrates tested or blank (no added carbohydrate) were prepared and 130 μL aliquots were dispensed into a 96-well plate. The washed isolates were diluted 10-fold in 1 mL of carbohydrate-free sMRS and 20 μL was used to inoculate each well. Experimental plates were pre-incubated anaerobically for 30 min, followed by the addition 50 μL of mineral oil (bioMérieux, Craponne, France) to each well. Immediately, the initial optical density (OD) at 595 nm was measured using a Multiscan Ascent 96-well plate reader (MTX Lab Systems, Vienna, VA, USA). The plates were incubated anaerobically at 37°C for 48 h. After 24 and 48 h incubation, the plates were vortexed to resuspend the cells and OD was recorded. Each well was blank-corrected at each time point (0, 24 and 48 h) and the maximum OD (ODmax) was calculated at 24 and 48 h. Utilization cutoffs were defined by the ODmax obtained and are as follows; low utilization: OD<0.4, moderate utilization: 0.4>OD<0.7 and high utilization: OD>0.7. The relative utilization of GOS and PDX was calculated to adjust for isolates incapable of growing in vitro.

#### Thin layer chromatography

Consumption of GOSP was determined by measuring the GOS fractions remaining in the post-fermentation supernatants of lactobacilli isolated from the IL and AC as previously described [39]. Briefly, 2 μL of a 1% (v/v) carbohydrate standard or bacterial post-fermentation supernatant was spotted onto TLC silica gel plates (Sigma-Aldrich, St. Louis, MO, USA). Glucose, galactose (Sigma-Aldrich), lactose (Fisher Scientific) and GOSP were included as carbohydrate standards. The TLC developing solvent was composed of a 2:1:1 mixture of n-propanol (Fisher Scientific), acetic acid (Fisher Scientific) and water. TLC plates were further prepared by spraying an ethanol based solution containing 0.5% (w/v) α-naphthol (Alfa Aesar) and 5% (v/v) H_2_SO_4_ (Sigma-Aldrich). TLC plates were visualized by heating at 100°C for 5 min.

### Culture-independent characterization of the microbiome

#### DNA extraction

Bacterial genomic DNA was isolated from 200–300 mg of IL and AC contents using a modified bead beating method [40,41]. The modifications included: (1) IL and AC content samples were lysed using a 3 min homogenization step with beads using a vortexer and (2) extracted DNA was resuspended in TE buffer overnight at 4°C instead of mechanical pipetting. These modifications were used to minimize DNA shearing for downstream applications. The DNA was purified using QIAmp DNA Stool Kits (Qiagen, Valencia, CA, USA). Extracted DNA was quantified by NanoDrop.

#### Quantitative PCR

Butyrate productivity was quantified from extracted bacterial genomic DNA from AC contents. Butyrate productivity was determined by amplification of the Butyryl-Coenzyme A transferase using previously published methods [42]. Standard curves of 10^2^ to 10^8^ CFU/mL were produced using a 573 bp gene product synthesized and ligated into a plasmid (GeneArt, Carlsbad, CA, USA). The minimum detectable CFU/mL was 10^3^. The AC bacterial genomic DNA was diluted 10-fold prior to quantification. Quantitative PCR was performed using the Applied Biosystems 7900HT Fast Real-Time PCR System (Applied Biosystems, Carlsbad, CA, USA). Each 10 μL qPCR reaction included 5 μL of 2x Power SYBR Green PCR Master mix (Applied Biosystems), bovine serum albumin at a final concentration of 1 μg/uL (New England Bio Labs, Ipswich, MA, USA), 0.5 μM of each primer and 2 μL of the DNA sample. The PCR conditions were 50°C for 2 min, 95°C for 10 min, followed by 40 cycles of 95°C for 15 s, 53°C for 1 min and 72°C for 30 s. Following amplification, a dissociation step was included to analyze the melting profile of the amplified products and ensure that no extraneous products were generated.

#### 16S amplicon sequencing

Genomic DNA isolated from IL and AC contents were analyzed by 16S amplicon sequencing at the Research and Testing Laboratory (RTL, Lubbock, TX) based upon RTL protocols [43]. Briefly, the V1-V3 hypervariable region of the 16S rRNA gene was amplified using the Gray28F (5’-GAGTTTGATCNTGGCTCAG-3’) and Gray519R (5’-GTNTTACNGCGGCKGCTG-3’) primer set. Sequencing was performed using the Roche 454 FLX instrument (Roche, Indianapolis, IN, USA). For a detailed description of the sequencing and bioinformatic analyses, refer to Ishak and colleagues [44]. Raw read data was submitted to the Sequence Read Archive (NCBI) and can be accessed with the accession number SRP049961.

#### Statistical analyses and illustrations

Analysis of piglet weight, weight gain, intestinal lengths and weights, intestinal histomorphology, fermentation products and bacterial enumeration was performed using the general linear model (GLM) procedure within SAS (Version 9.2, SAS Institute, Cary, NC, USA). When significant comparisons (P<0.05) were observed, post hoc analysis using the least significant difference (LSD) was used. Lactobacilli communities are presented as stacked bar charts produced with R [45] using the gplots [46] package. Dendrograms were constructed using the Bray-Curtis Dissimilarity index using the vegan [47] R package. Prior to analysis of the 16S amplicon sequencing data, rarefaction was used to standardize richness to 3118 sequences per sample, thus facilitating comparisons among groups. In addition, IL samples were analyzed separately from AC samples. Microbial diversity and richness was quantified as the number of operational taxonomic units (OTUs) or with Chao1's richness estimator (each were defined based on 3% divergence). The impact of diet on bacterial diversity and total estimated richness was evaluated using one-way ANOVA. The overall variability within the microbiota was illustrated using Principle Coordinates Analysis (PCoA), and multivariate differences among diet groups were evaluated using distance-based redundancy analysis (dbRDA) [48]. For PCoA and dbRDA, phylogenetic distances among samples were first calculated using the UniFrac distance measure, unweighted UniFrac distance considers only changes in composition (i.e., identities) [49]. UniFrac was calculated using QIIME [50], and all other analyses were conducted in R [45], using the vegan [47] and labdsv [51] packages. Individual bacterial taxa were screened for differences among diets using ANOVA. Prior to analysis, relative abundances were transformed using an arcsin transform (p’ = arcsin (√p)). To control for errors due to multiple testing and to limit the amount of false positives, ANOVA model p-values were adjusted to maintain a false discovery rate (FDR) of 10%. Indicator species analysis [52] was used to identify individual species within the genus *Lactobacillus* that were indicative of each of the diets. Indicator species analysis synthesizes information about occurrence and abundance of individual taxa, and this information is summarized as an indicator score. The analysis also provides a randomization test of the degree to which taxa are indicative of a particular state. Here, all species are shown, regardless of the results of the randomization test.

## Results

### Animal observations

The final body weight and overall weight gain of the piglets were not different between the groups (P>0.05, data not shown). Additionally, the total intestinal weight and length did not differ between groups and averaged 196 ± 41.3 g/kg and 148.8 ± 40.3 cm/kg; respectively. Intestinal histomorphology demonstrated that the ileal (IL) villus length, crypt depth and ascending colon (AC) cuff width were not significantly different among the groups (P>0.05; Table 1). However, the SOW group had significantly deeper AC cuff depth as compared to FORM and F+GP groups (P<0.0001). While there were no differences in the percentage of dry matter in the AC contents (P>0.05, Table 1), the percentage of dry matter in the IL was lower in the F+GP group than the SOW group (P = 0.04), but not the FORM group.

**Table 1:** Histomorphology of ileum and ascending colon of 21-d-old piglets fed formula (FORM), formula supplemented with GOS and PDX (F+GP) or sow-reared (SOW).

	Diets						
	FORM		F+GP		SOW		GLM ANOVA
	Mean	SD	Mean	SD	Mean	SD	p-value
Ileum							
Villus Length (μm)	495	156	401	61	387	70	> 0.05
Crypt Depth (μm)	184	45	177	30	202	32	> 0.05
Dry Matter (%)	12.6^ab^	3.6	9.9^a^	2.9	15.3^b^	5.7	0.04
Ascending Colon							
Cuff Width (μm)	51^b^	5	58^a^	6	56^ab^	5	0.05
Cuff Depth (μm)	275^a^	24	261^a^	24	357^b^	38	< 0.0001
Dry Matter (%)	21.3	3.9	21.0	3.0	20.5	5.2	> 0.05

^a b^ values within the row with the same letters are not different.

### Isolation and characterization of intestinal lactobacilli

Based on published research, we hypothesized that supplementation of GOS/PDX should selectively stimulate the growth of lactobacilli. Therefore immediately following euthanasia, IL and AC contents were plated on *Lactobacillus* selective (LBS) agar to obtain lactobacilli isolates. Total presumptive *Lactobacillus* counts (CFU/g) were not significantly different between the groups or location (P>0.05, data not shown). While the absolute abundance of lactobacilli was not different between groups, it may be the composition of the lactobacilli community that is important. Therefore, approximately 10 isolates per piglet were cultivated and speciated using a combination of the HSP60 and 16S rDNA gene sequences. All of the isolates identified were of the order *Lactobacilliales* and were represented by eight *Lactobacillus* species and two non-*Lactobacillus* genera. Unfortunately, the co-isolation of *Pediococcus* and *Weisella* with *Lactobacillus* in the formula-fed piglets was quite significant (range 10–37% of total isolates). The *Pediococcus* and *Weisella* isolates were removed from further analyses.

**Fig 1:**
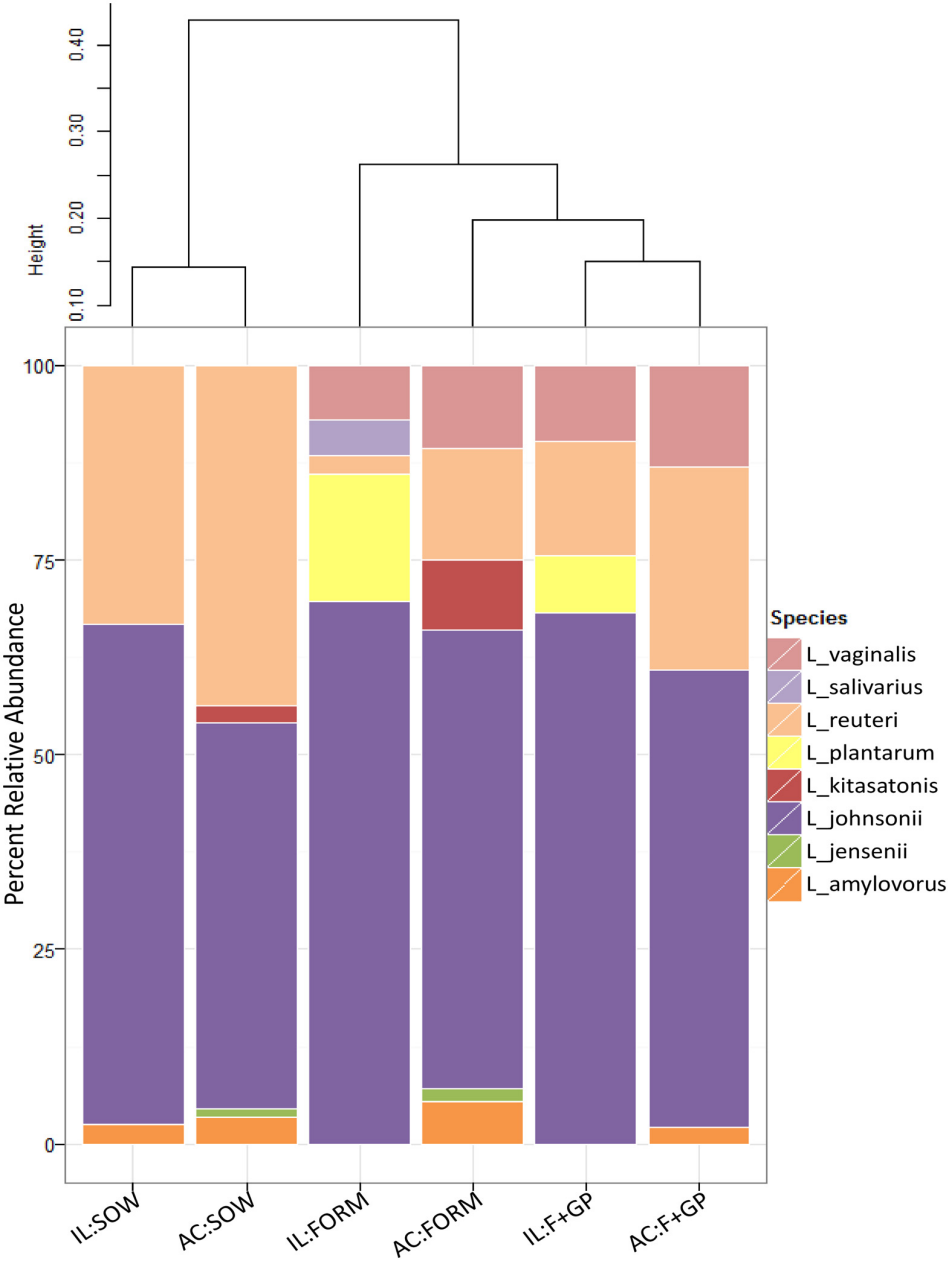
Lactobacilli population is dominated by few members. Lactobacilli communities isolated from the ileum (IL) and ascending colon (AC) of 21 d old piglets fed either a bovine milk-based formula (FORM), formula supplemented with GOS and PDX (F+GP) or sow-reared (SOW). Stacked bar chart represents the percent relative abundance of each lactobacilli detected in the community. Beta diversity was calculated using the Bray-Curtis Dissimilarity Index and is visualized by a dendrogram.

Culture-dependent analysis showed a variable lactobacilli community between groups (Fig 1). The SOW group was dominated by two lactobacilli: *L*. *johnsonii* (IL: 64%, AC: 50%) and *L*. *reuteri* (IL: 33%, AC: 44%). While *L*. *johnsonii* was also highly abundant in the FORM and F+GP groups, these two groups had a more diverse lactobacilli community as compared to the SOW group. *L*. *vaginalis* was solely detected in the FORM and F+GP groups, representing 7–13% (IL and AC) of the isolates.

**Fig 2:**
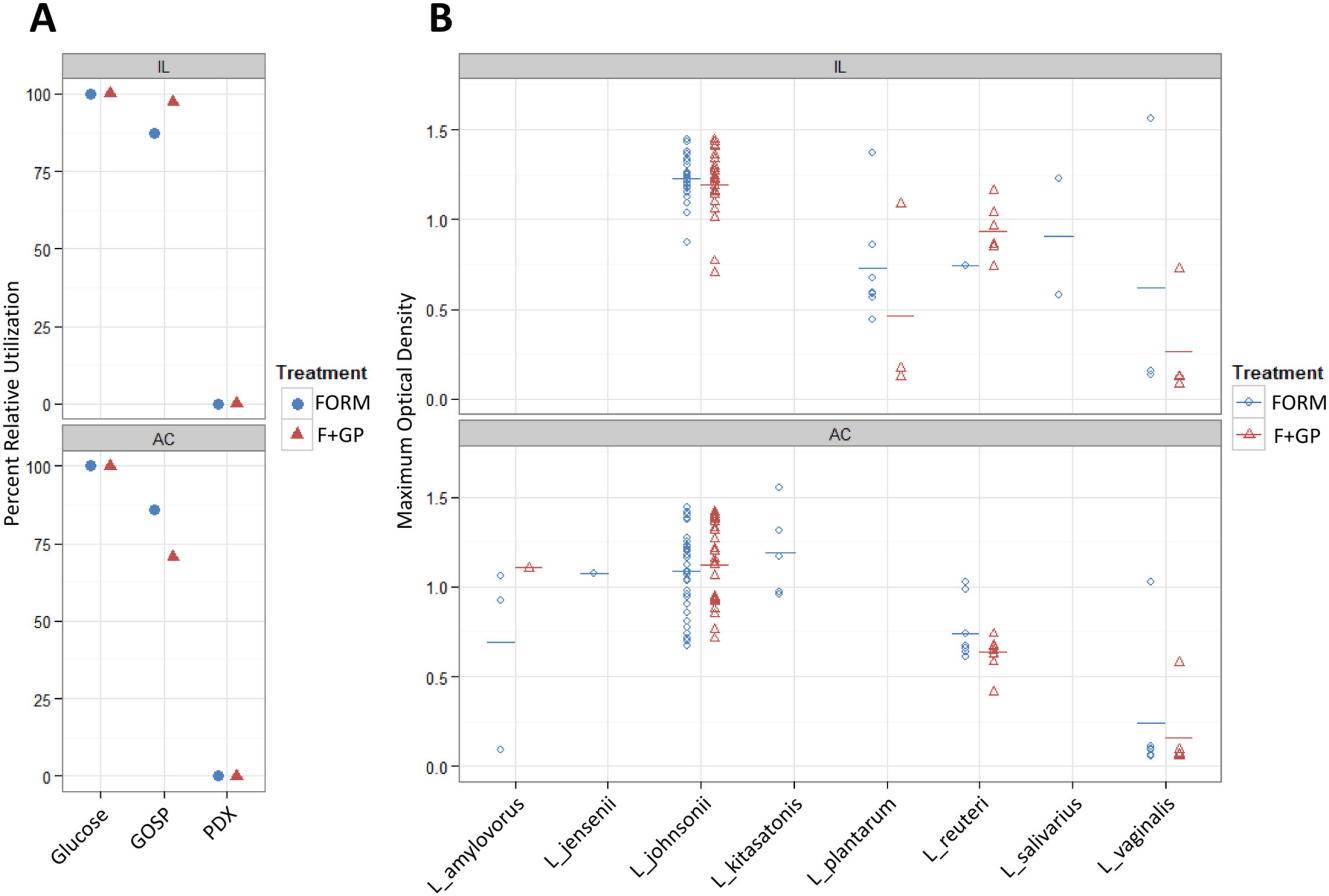
Several isolated lactobacilli identified as GOS utilizers. Carbohydrate utilization of lactobacilli communities isolated from the ileum (IL) and ascending colon (AC) of 21 d old piglets fed either a bovine milk-based formula (FORM), formula supplemented with GOS and PDX (F+GP). (A) Percent relative utilization (OD>0.7) of glucose, Purimune galactooligosaccharide (GOSP) or PDX. To account for isolates not capable of *in vitro* fermentation, utilization was normalized to maximum number of isolates capable of utilizing glucose as a sole carbon source. (B) Maximum optical density (595 nm) of *Lactobacilli* sp. when GOSP is supplemented as a sole carbon source. Colored bar indicates the mean maximum optical density. Not all *Lactobacillus* sp. were detected in each treatment or location.

To determine if the supplementation of GOS/PDX selectively enriches for a GOS/PDX fermenting community, carbohydrate utilization was conducted with the FORM and F+GP lactobacilli isolates (Fig 2 and S1 Table). The majority of lactobacilli isolates from both the FORM and F+GP piglets were found to utilize GOS well (ΔOD>0.7); whereas, the utilization of PDX was minimal. While a larger proportion of lactobacilli isolates were defined as high utilizers of GOS in the IL (FORM: 87.5% vs. F+GP: 97.3%), this was not the case in the ascending colon (FORM: 86% vs. F+GP: 70.7%). Thin layer chromatography was conducted to determine if the ability to consume different GOS fractions was different due to GOS supplementation (data not shown). In general, the lactobacilli isolates with a low (ΔOD<0.4) or moderate (0.4>ΔOD<0.7) ability to utilize GOS corresponded with a reduction in the mono- and disaccharide fractions (DP1-2). The lactobacilli isolates classified as high utilizers (ΔOD>0.7) demonstrated partial consumption of trisaccharide GOS (DP3) as well as complete consumption of the DP1-2 fractions. We did not observe any reduction in the longer GOS chains (DP4-6) by any of the isolates. Interestingly, *L*. *vaginalis* was observed to differentially utilize GOS *in vitro*. The few *L*. *vaginalis* isolates that grew to an OD>0.4 showed at least partial consumption of GOS (DP1-3); whereas, the majority of isolates were not capable of fermenting GOS as a sole carbon source. Overall, the consumption of GOS was not different between the FORM and F+GP piglets regardless of location.

### Characterization of intestinal bacterial community

The IL and AC microbiota was analyzed by 16S amplicon sequencing which generated a total of 292,547 sequences with an average of 5,519 sequences per sample. Following sequence normalization, overall microbial diversity was determined by calculating the total number of operational taxonomic units (OTUs) detected and total richness was estimated by the Chao1 index (Table 2). The SOW group had higher IL microbial diversity and total richness as compared to the formula-fed piglets (OTUs: P = 0.01, Chao1: P = 0.025; respectively). The microbial diversity and total richness were not significantly different in the AC.

**Table 2:** Microbial analysis from ileal and ascending colon contents of 21d old piglets fed formula (FORM), formula supplemented with GOS and PDX (F+GP) or sow-reared (SOW) as determined by 16S amplicon sequencing.

	Diets						
	FORM		F+GP		SOW		GLM ANOVA
	Mean	SD	Mean	SD	Mean	SD	p-value
Ileum							
Total Sequences	9597	2331	7973	1823	5154	2029	ND^2^
OTUs^1^ (97%)	667^a^	157	660^a^	150	851^b^	146	0.01
Chao1 (97%)	1266^a^	265	1322^a^	263	1647^b^	300	0.025
Ascending Colon							
Total Sequences	5067	1529	4674	912	3457	1293	ND^2^
OTUs^1^ (97%)	892	80	902	95	868	153	> 0.05
Chao1 (97%)	1680	190	1686	176	1757	240	> 0.05

^1^ OTU and Chao1 normalized to 3118 sequences per sample.

^2^ ND = not determined.

^a b^ values within the row with the same letters are not different.

**Fig 3:**
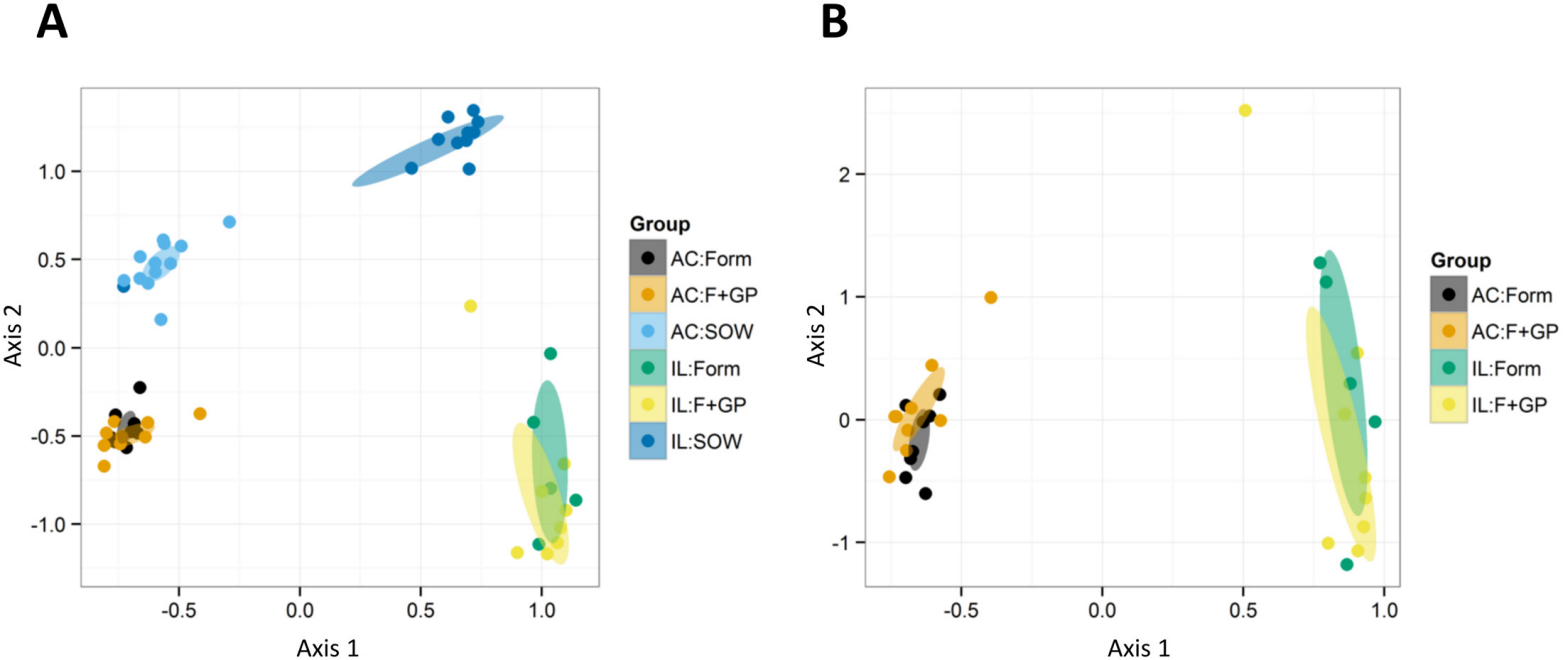
Bacterial communities vary greatly by location, but less by diet. Ileal (IL) and ascending colon (AC) microbiome of 21 d old piglets fed either a bovine milk-based formula (FORM), formula supplemented with GOS and PDX (F+GP) or sow-reared (SOW) illustrated by Principle Coordinate Analysis (PCoA). PCoA was constructed from the unweighted Unifrac distance measure which only considers the change in composition not abundance. PCoA of the 16S amplicon sequencing data set: (A) all three treatments; (B) formula-fed piglets only.

The overall variation in the piglet microbiota among the three groups was evaluated using a distance-based redundancy analysis (dbRDA). Distances were calculated using the unweighted Unifrac distance measure and were illustrated using Principle Coordinates Analysis (PCoA). The PCoA of the piglet microbiome shows a clear divergence in location of samples (IL vs. AC) and between the SOW piglets and the formula-fed piglets (Fig 3a). This divergence was evident following characterization at the taxonomic level. A heatmap was constructed with the relative abundances of the dominant taxa in both locations (S1 Fig). Groups were clustered using the unweighted Unifrac distances. In the IL, the SOW group was characterized by a high abundance of *Clostridium* spp. whereas the formula-fed piglets were defined more by the presence of *Weissella* spp. In the AC, the differences in relative abundance of *Faecalibacterium* and *Parabacteroides* showed separation of the SOW group from the formula-fed groups (S2 Fig).

In general, our data (Fig 3 and S1 and S2 Figs) demonstrates that sow-reared piglets may not be an appropriate reference group. The primary focus of this study was to analyze the bacterial composition due to a prebiotic supplementation. The differences between the formula-fed piglets and the sow-reared piglets were not solely dietary; therefore, the 16S amplicon sequencing data was reanalyzed without the SOW group in order to observe differences due solely to the prebiotic supplementation.

Consistent with the previous 16S amplicon sequencing data, the microbial diversity and total richness of the FORM and F+GP groups were not significantly different in the IL or AC (P>0.05, data not shown). Similar clustering between the FORM and F+GP groups by location was observed when determining the overall variation of the microbiome with a PCoA of the unweighted Unifrac distances (Fig 3b). In the IL, no detected genera had a FDR less than 10%; therefore further statistical analysis was not performed. However, six genera were detected in the AC that had an FDR less than 10% and were determined to be significantly different (P<0.05) between the FORM and F+GP groups based on differences in relative abundance (S2 Table and S2 Fig). *Ruminococcus*, *Oscillopira*, *Hydrogenoanaerbacterium* and *Catabacter* were significantly more abundant in the FORM group, while *Parabacteroides* and *Lactobacillus* were significantly more abundant in the F+GP group. Interestingly, the relative abundance of *Parabacteroides* more than two times greater in the F+GP piglets (FORM: 8.67 ± 2.22 vs. F+GP: 18.33 ± 3.98; S2 Table).

### Culture-independent examination of lactobacilli community

**Fig 4:**
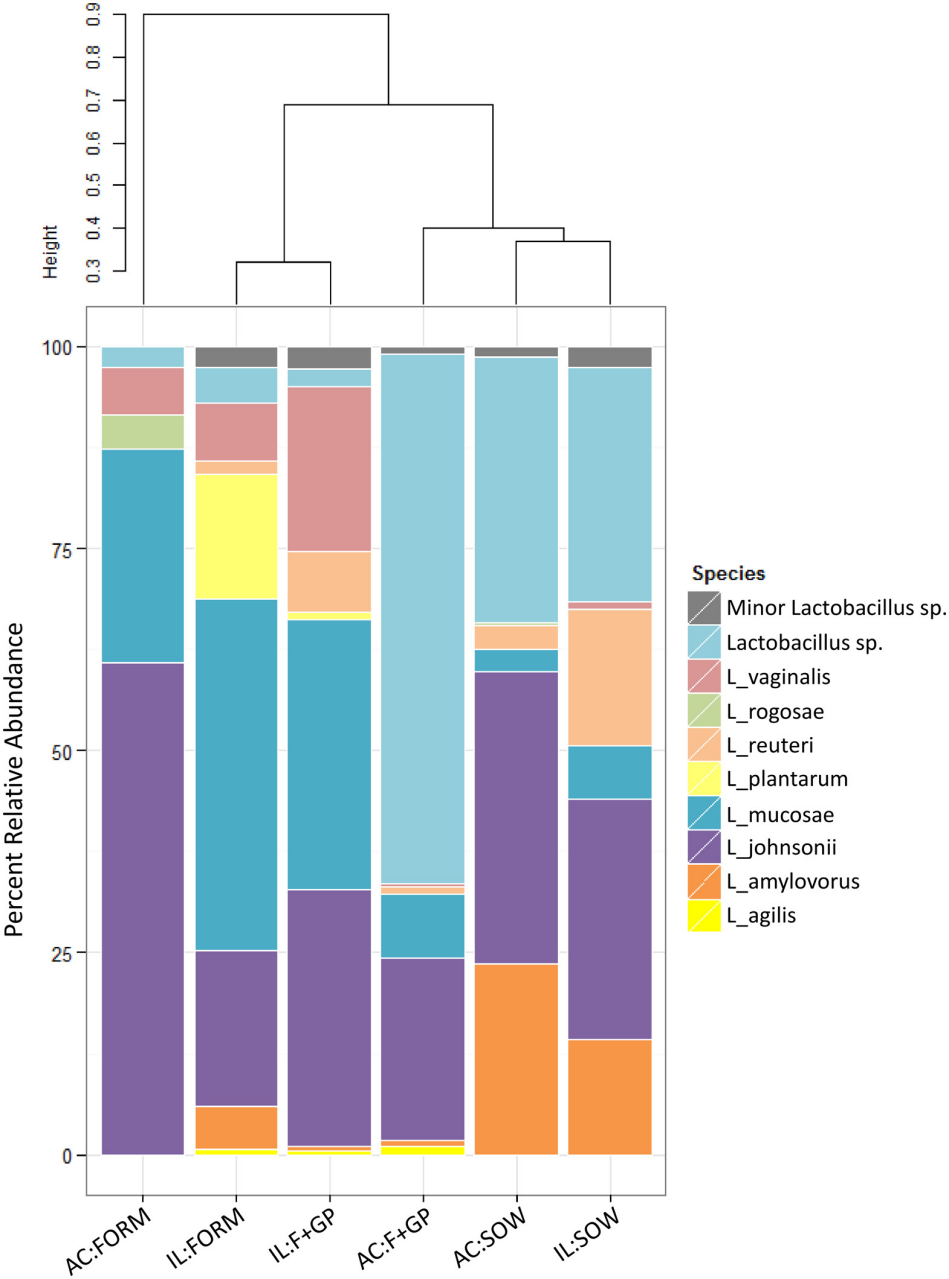
Culture-independent analysis reveals a more diverse lactobacilli community. Lactobacilli communities from the ileum (IL) and ascending colon (AC) of 21 d old piglets fed either a bovine milk-based formula (FORM), formula supplemented with GOS and PDX (F+GP) or sow-reared (SOW) as determined by 16S amplicon sequencing. Stacked bar chart represents the percent relative abundance of each lactobacilli detected in the community. Beta diversity was calculated using the Bray-Curtis Dissimilarity Index and is visualized by a dendrogram. Lactobacilli represented by the label Lactobacillus sp. were unable to be assigned a species. Minor Lactobacillus sp. is an aggregate of lactobacilli present in > 1% percent relative abundance (S3 Table).

16S amplicon sequencing analysis identified that the *Lactobacillus* genus differed between the FORM and F+GP groups in the AC (SOW group is included for reference). Eight major species of *Lactobacillus*, having a relative abundance above 1% of the total lactobacilli community, were detected in IL and AC (Fig 4). Consistent with the culture-dependent results, *L*. *johnosonii* is a dominant member of the lactobacilli community in all piglets. On the other hand, *L*. *mucosae* was detected in all piglets by 16S amplicon sequencing; however, was not among the lactobacilli isolated from either the IL or AC.

**Fig 5:**
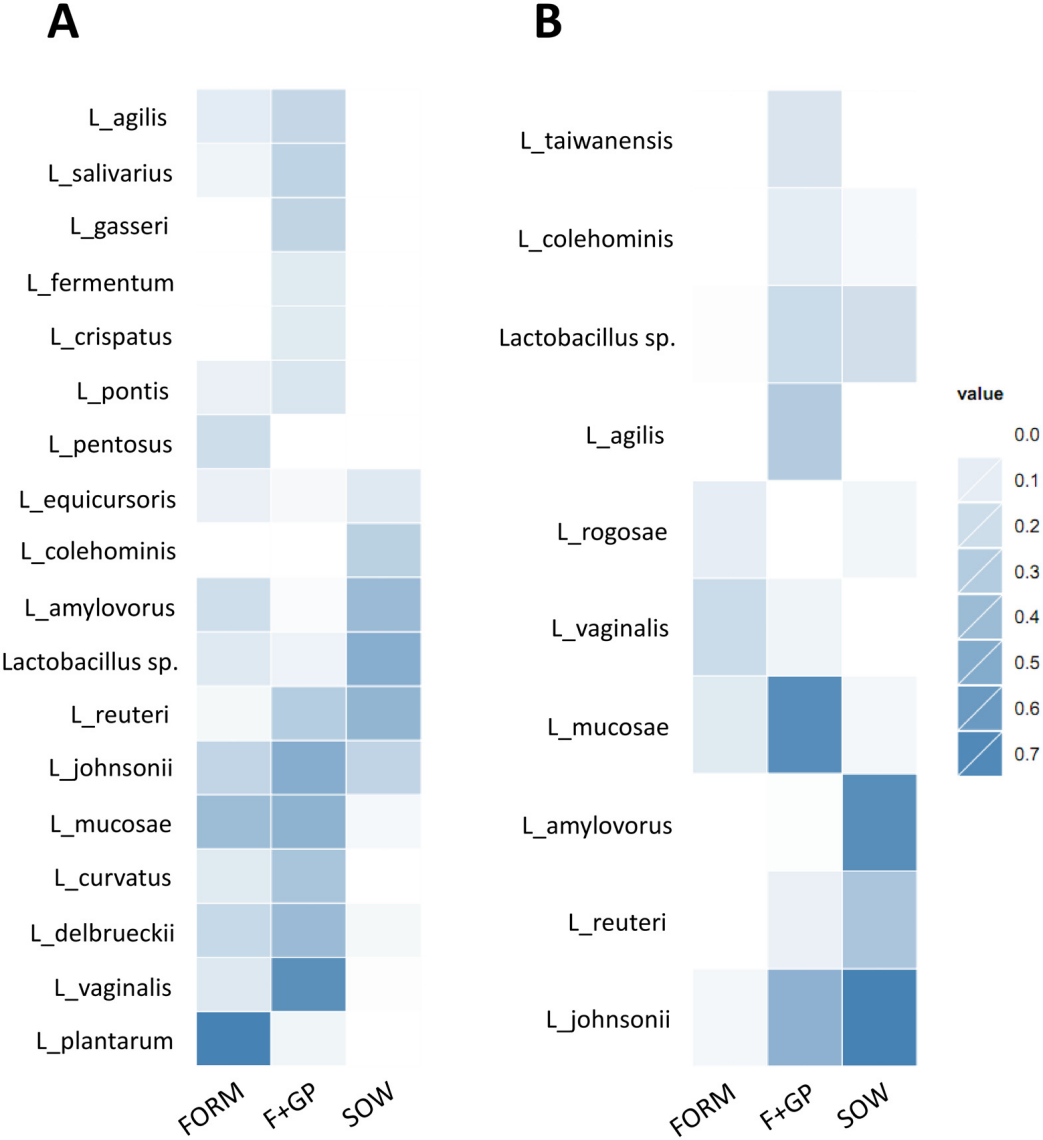
Identification of the predominant *Lactobacillus* spp. in each group. Indicator species analysis (ISA) of the lactobacilli community of 21 d old piglets fed either a bovine milk-based formula (FORM), formula supplemented with GOS and PDX (F+GP) or sow-reared (SOW, included for reference). Color change demonstrates the more a species is indicative of a diet. The higher the indicator score (i.e. darker color), the more that species is indicative of that group. ISA scores for the genera *Lactobacillus* detected in the (A) IL; (B) AC.

Further analysis was conducted to determine if any lactobacilli were predictive of a particular treatment (Fig 5a and 5b). Indicator species analysis showed that *L*. *plantarum*, *L*. *amylovorous* and *L*. *pentosus* to be indicative of the group FORM but not the F+GP group in the IL (Fig 5a). Conversely, *L*. *vaginalis*, *L*. *johnsonii* and *L*. *reuteri* were more indicative of the F+GP group in the IL. In the AC, *L*. *johnsonii*, *L*. *mucosae*, *L*. *agilis* and *L*. *reuteri* were highly indicative of the F+GP group as compared to the FORM group; whereas, *L*. *vaginalis* and *L*. *rogosae*, were slightly indicative of the FORM group (Fig 5b).

### Fermentation products

**Fig 6:**
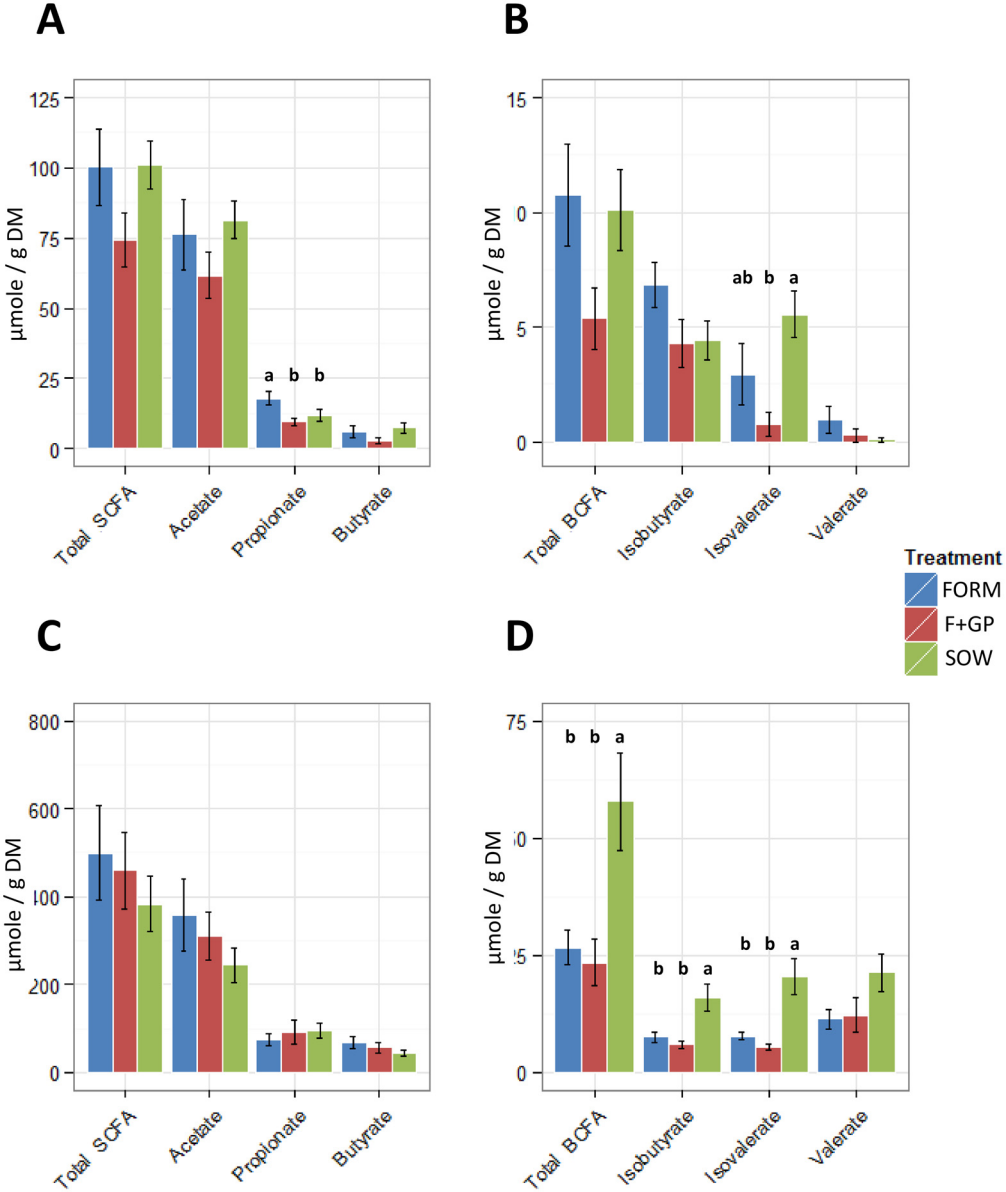
Prebiotic supplementation leads to minimal changes in static volatile fatty acid concentration. Volatile fatty acids detected in ileal (IL) and ascending colon (AC) contents of 21 d old piglets fed either a bovine milk-based formula (FORM), formula supplemented with GOS and PDX (F+GP) or sow-reared (SOW). VFAs detected in IL: (A) Short-chain fatty acids; (B) Branch-chain fatty acids. VFAs detected in the AC: (C) Short-chain fatty acids; (D) Branch-chain fatty acids. Values with different superscripts are significantly different (p<0.05).

Volatile fatty acids (VFA) were determined from the IL and AC contents (Fig 6). In the IL, propionate was higher (P = 0.028) in the FORM group as compared to the F+GP and SOW groups; (Fig 6a) and isovalerate concentration was lower (P = 0.004) in the F+GP group when compared to the SOW group, but not the FORM group (Fig 6b). The IL pH ranged from 7.7–7.8 and was not different among the treatment groups (P = 0.892). In the AC, there were no significant differences in the measured SCFAs (P>0.05, Fig 6c). Furthermore, the SOW group had higher observed levels of isobutyrate (P = 0.004), isovalerate (P = 0.001) and total BCFA (P = 0.008) than the formula-fed groups (Fig 6d). The AC pH tended (P = 0.055) to be lower in both formula-fed groups (FORM: 6.8, F+GP: 6.6) than in the SOW group (7.3).

The total butyrate productivity was evaluated in the AC contents using quantitative PCR. The absolute abundance of the butyryl-Coenzyme A transferase gene was not significantly different between the three groups (P = 0.4281, data not shown).

## Discussion

Prebiotic supplementation of infant formula is well tolerated, improves the consistency and frequency of stools and provides a bifidogenic effect [14,20–22,53–57]. Additionally, our group has shown that prebiotic supplementation does not increase bacterial translocation in neonatal piglets [32]. Taken together, these studies demonstrate the nutritional benefits and safety for prebiotic supplementation of infant formula. However, a detailed examination of the impact of prebiotics on the composition of the gut microbiome and the products of microbial fermentation is lacking. In addition, identification of prebiotic-responsive lactobacilli could assist in determining which organisms are enhanced by prebiotic supplementation. In this study, the impact of GOS and PDX (2 g/L each) provided in a bovine milk-based infant formula was examined in neonatal piglets, a relevant infant model [26–28]. Since prebiotic fermentation is restricted to the distal GI tract [58], the IL and AC were targeted for analysis.

Prebiotic supplementation did not significantly affect the body weight gain or intestinal weight or length compared to SOW or FORM. Formula feeding minimally impacted the intestinal histomorphology, compared to SOW with no impact of prebiotic supplementation. The AC crypt depth was significantly deeper in the SOW piglets as compared to the formula-fed piglets, which is consistent with our previous study that investigated bacterial translocation of GOS/PDX-supplemented formula-fed piglets [32]. Another study that supplemented formula with inulin and GOS did not detect differences in colonic histomorphology due to prebiotic supplementation; however a sow-reared group was not included [59]. Additional piglet studies that measured intestinal histomorphology following prebiotic supplementation only reported ileal measurements [24,31]. Therefore, we can conclude that the addition of GOS and PDX to formula is well tolerated and minimally impacts piglet intestinal development.

Changes in the IL and AC microbiota due to prebiotic supplementation were evaluated by high-throughput 16S amplicon sequencing. Significant differences in the intestinal microbiota composition were observed between the SOW and formula-fed piglets. In this study, there were differences in nutrition (sow’s milk versus bovine milk-based formula), housing (group housing with siblings and sow versus individual-housing of formula-fed piglets) and exposure to novel microbiota. Previous work has shown that formula-fed and breast-fed infants have different microbial composition [7,60], that stress can affect the microbial composition [61] and that homogenization of the gut bacterial community may occur in co-housed animals [62]. Therefore, future microbial studies examining formula-fed versus mother-fed animals should take care to minimize the variation between groups.

Due to the distinct differences between SOW and the formula-fed piglets, the 16S amplicon sequencing data was reanalyzed without SOW in order to isolate differences solely due to prebiotic supplementation. The bacterial diversity and richness did not differ between FORM and F+GP; however, the relative abundance of several bacterial genera were significantly different in the AC. Interestingly, *Parabacteroides* and *Lactobacillus* were identified as being significantly more abundant in the AC of F+GP piglets. *Parabacteroides*, closely related to *Bacteroides* [63], may possess enzymes capable of utilizing either GOS or PDX; however, there is currently no published data to confirm this hypothesis. Martinez and colleagues [64] did report a significant increase of *Parabacteroides distasonis* in human feces following consumption of resistant starch type 4 but not resistant starch type 2. Future studies should evaluate the *Parabacteroides* community in relation to GOS/PDX supplementation in greater detail.

Historically, lactobacilli are considered health promoting microorganisms and have recently gained notoriety as probiotics [65]. Additionally, *Lactobacillus* sp. have been shown to be stimulated by prebiotic inclusion in formula milk [23,24]. Therefore, we hypothesized that the supplementation of GOS/PDX may alter the *in vivo* lactobacilli community. Following speciation of presumptive lactobacilli isolates, we observed that *L*. *johnsonii* was the predominant member in the IL and AC of all piglets. While the formula-fed piglets had a more diverse lactobacilli community, it was not apparent if any *Lactobacillus* sp. isolated were prebiotic-responsive. Therefore, the lactobacilli isolates were tested for their ability to utilize GOS and/or PDX as a sole carbon source (direct fermentation). We observed extensive utilization of GOS but minimal utilization of PDX by *Lactobacillus* sp. isolated from the IL and AC of all formula-fed piglets. Therefore, we further hypothesized that GOS supplementation may select for lactobacilli isolates that are capable of consuming longer chain GOS (DP3-6) rather than the mono- and disaccharide fractions (DP1-2) that are mostly removed by digestion prior to the IL and AC. To test this hypothesis, the post-fermentate was analyzed by TLC for consumption of GOS fractions (DP1-6). In general, most lactobacilli isolates were capable of consuming the mono- and disaccharide fractions (DP1-2); however, a smaller fraction of lactobacilli were capable of consuming trisaccharide GOS (DP3). Importantly, there was no difference regarding the number of FORM and F+GP lactobacilli isolates that could ferment the longer GOS fractions. Direct fermentation of GOS or PDX doesn’t appear to be responsible for the increase in the lactobacilli population in the AC of the F+GP piglets.

Consistent with culture-dependent analysis, *L*. *johnsonii* was highly abundant in both FORM and F+GP piglets and was indicative of the F+GP treatment in the IL and AC. Various strains of *L*. *johnsonii* have been investigated in depth for their health promoting properties. In a rodent model for diabetes mellitus type 1 (T1DM), *L*. *johnsonii* was differentially abundant in the T1DM-resistant rats versus their T1DM-prone cohorts [66]. Furthermore, transmission of the strain *L*. *johnsonii* N6.2 can delay the onset of T1DM in prone rats [67]. The potential mechanisms of action of *L*. *johnsonii* N6.2 in this rodent model have been investigated and include enhancement of intestinal barrier function [67], reduction of intestinal oxidative stress [67] and modulation of the immune system [68–70]. Administration of *L*. *johnsonii* (La-1) to mice with branchial induced asthma dampened the autoimmune response by reducing the proportion of CD4^+^ T lymphocytes expressing IL-4 and increasing CD4^+^ T lymphocytes expressing IFNγ [71]. Thus, promoting the growth of *L*. *johnsonii* by feeding PDX and GOS has the potential to modulate immune development; however, this remains to be directly tested.

Indicator species analysis of the 16S amplicon sequencing data identified two additional *Lactobacillus* species that were characteristic of the F+GP piglets. *L*. *vaginalis* was strongly indicative of the F+GP piglets in the IL, whereas *L*. *mucosae* was strongly indicative of the F+GP piglets in the AC. Surprisingly, the majority of *L*. *vaginalis* representative isolates (79%) were not GOS utilizers *in vitro* suggesting that direct GOS utilization did not lead to an increase in *L*. *vaginalis in vivo*.

Commonly, changes in microbial communities are coupled with the measurement of select byproducts of fermentation. Prebiotics, resistant to host digestion in the proximal GI of piglets and humans, are selectively fermented by the microbial community in the distal GI where the primary metabolites produced are SCFA from carbohydrate fermentation, [72] and BCFA from protein fermentation [73,74]. In the IL, no increase in total SCFA due to the prebiotics was detected; F+GP had the lowest SCFA concentration. Mono- and disaccharides are the primary substrate responsible for SCFA production in the IL, whereas prebiotics are largely fermented in the large bowel [58]. Total SCFA concentrations were not significantly different among the three groups in the AC; however, it is estimated that >95% of SCFA are rapidly absorbed by colonocytes [58]. Without the ability to measure the rate of host absorption of SCFA, and the total amount of SCFA in AC contents, we cannot determine the degree to which GOS and PDX were fermented *in vivo*. In young pigs fed a transgalactooligosaccharide-supplemented casein-cornstarch diet [75], ileal SCFA concentrations were similar to the amounts observed in the current study. Additionally, Fava and colleagues [76] reported that PDX supplementation did not affect SCFA in the distal small intestine or proximal colon of piglets. Thus, our results are consistent with these studies. The production of BCFA is primarily due to the fermentation of amino acids rather than prebiotics [73,74]. The SOW group had significantly higher concentrations of isobutyrate, isovalerate and total BCFA. This is consistent with additional piglet studies performed in our group [77], and may be due to proteins in sow milk that are resistant to small intestinal digestion, such as immunoglobulins.

This study provided a descriptive analysis of changes in the intestinal communities of neonatal piglets fed formula milk supplemented with GOS and PDX. Most interestingly were the significant increases in *Parabacteroides* and *Lactobacillus* sp. in the F+GP piglets. We did not observe an increase in the amount of lactobacilli that can utilize GOS nor the DP of GOS that was consumed. Therefore, we conclude that direct utilization of GOS was not responsible for the observed increase in *Lactobacillus* sp. Additional studies should be conducted to determine if GOS/PDX utilization by *Parabacteroides* sp. *in vitro* and to identify if this genus has physiological benefits to a neonatal piglet.

## Supporting Information

S1 Fig Ileal microbial communities of 21-d-old piglets fed either a bovine milk-based formula (FORM), formula supplemented with GOS and PDX (F+GP) or were sow-reared (SOW). Heatmap illustrates the relative abundances of top 20 genera detected in the ileal contents. Hierarchical clustering of unweighted Unifrac distances is represented by the dendrogram.
(TIFF)

S2 Fig Ascending colon microbial communties of 21-d-old piglets fed either a bovine milk-based formula (FORM), formula supplemented with GOS and PDX (F+GP) or were sow-reared (SOW). Heatmap illustrates the relative abundances of top 35 genera detected in ascending colon contents. Hierarchical clustering of unweighted Unifrac distances is represented by the dendrogram.
(TIFF)

S1 Table Carbohydrate utilization of the lactobacilli isolated from piglets fed formula (FORM), formula supplemented with GOS and PDX (F+GP). Values represent the percent of lactobacilli able to utilize either glucose, GOS or PDX as a sole carbon source.
(DOCX)

S2 Table Genera of ascending colon contents of 21d old piglets fed formula (FORM), formula supplemented with GOS and PDX (F+GP). Genera listed were significantly different between the FORM and F+GP piglets.
(DOCX)

S3 Table Lactobacilli detected in ileum and ascending colon of 21d old piglets fed formula (FORM), formula supplemented with GOS and PDX (F+GP) or sow-reared (SOW) as determined by 16S amplicon sequencing. Values represent the relative abundance of *Lactobacilli* sp.
(DOCX)
