# Supplementary figures and images for: Characterization of the Intestinal Lactobacilli Community following Galactooligosaccharides and Polydextrose Supplementation in the Neonatal Piglet
Source: PLoS One. 2015 Aug 14;10(8):e0135494. doi: 10.1371/journal.pone.0135494 (PMC4537252; doi:10.1371/journal.pone.0135494)

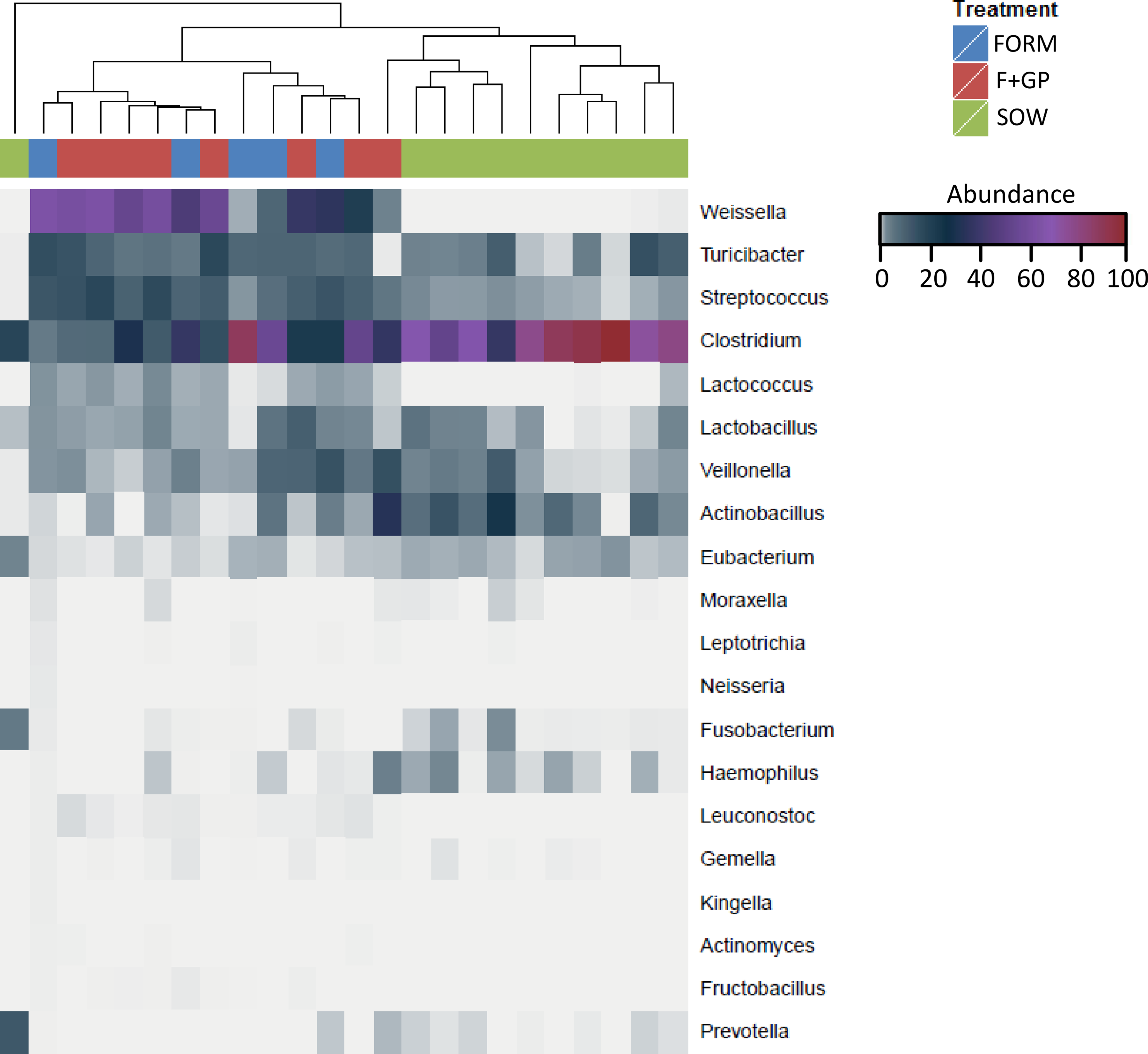

Supplement: S1 Fig — Heatmap illustrates the relative abundances of top 20 genera detected in the ileal contents. Hierarchical clustering of unweighted Unifrac distances is represented by the dendrogram. (TIFF) [file pone.0135494.s001.tiff]

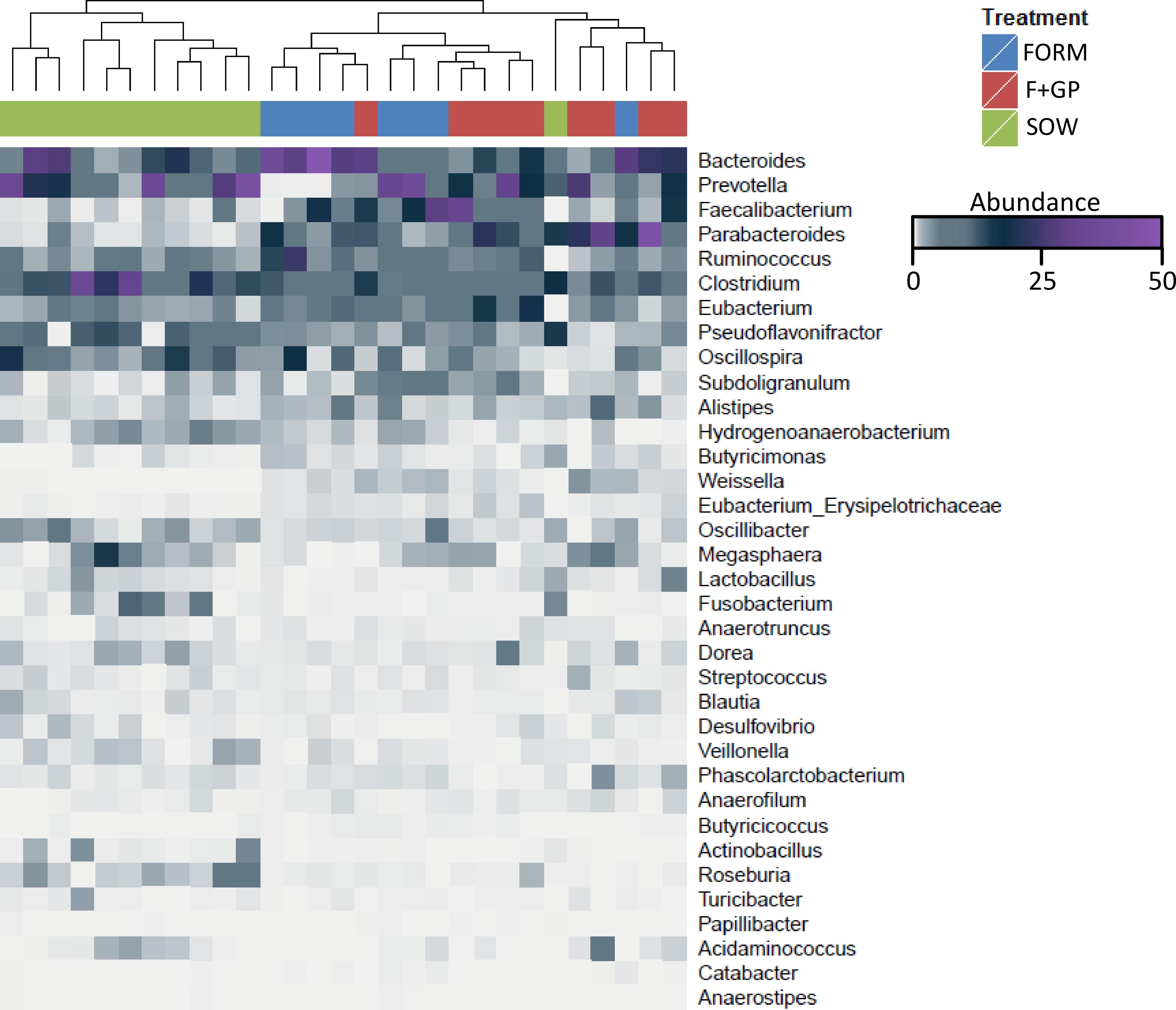

Supplement: S2 Fig — Heatmap illustrates the relative abundances of top 35 genera detected in ascending colon contents. Hierarchical clustering of unweighted Unifrac distances is represented by the dendrogram. (TIFF) [file pone.0135494.s002.tiff]
