# Supplementary material for: Characterization of the Intestinal Lactobacilli Community following Galactooligosaccharides and Polydextrose Supplementation in the Neonatal Piglet
Source: PLoS One. 2015 Aug 14;10(8):e0135494. doi: 10.1371/journal.pone.0135494 (PMC4537252; doi:10.1371/journal.pone.0135494)
Supplement: S1 Table — Values represent the percent of lactobacilli able to utilize either glucose, GOS or PDX as a sole carbon source. (DOCX) [file pone.0135494.s003.docx]

Table S1. Carbohydrate utilization of the lactobacilli isolated from piglets fed formula (FORM), formula supplemented with GOS and PDX (F+GP).^1^

|  | | FORM | | |  | F+GP | | |
| --- | --- | --- | --- | --- | --- | --- | --- | --- |
|  | | Glucose | GOS | PDX |  | Glucose | GOS | PDX |
| Ileum | |  |  |  |  |  |  |  |
|  | Low Utilization^2^ | 2.3 | 4.7 | 51.2 |  | 7.3 | 12.2 | 58.5 |
|  | Moderate Utilization | 4.7 | 14.0 | 48.8 |  | 2.4 | 0.0 | 41.5 |
|  | High Utilization | 93.0 | 81.4 | 0.0 |  | 90.2 | 87.8 | 0.0 |
|  | Relative Utilization  to Glucose^3^ | 100 | 87.5 | 0.0 |  | 100 | 97.3 | 0.0 |
| Ascending Colon | |  |  |  |  |  |  |  |
|  | Low Utilization | 10.7 | 10.7 | 89.3 |  | 10.9 | 10.9 | 84.8 |
|  | Moderate Utilization | 0.0 | 12.5 | 10.7 |  | 0.0 | 26.1 | 15.2 |
|  | High Utilization | 89.3 | 76.8 | 0.0 |  | 89.1 | 63.0 | 0.0 |
|  | Relative Utilization  to Glucose | 100 | 86 | 0.0 |  | 100 | 70.7 | 0.0 |

^1^ Data presented as percent utilizers

^2^ Utilization defined as low: OD<0.4, moderate: 0.4>OD<0.7 and high: OD>0.7

^3^ Percent of isolates capable of utilization of glucose *in vitro* under the chosen conditions
