# Supplementary material for: Characterization of the Intestinal Lactobacilli Community following Galactooligosaccharides and Polydextrose Supplementation in the Neonatal Piglet
Source: PLoS One. 2015 Aug 14;10(8):e0135494. doi: 10.1371/journal.pone.0135494 (PMC4537252; doi:10.1371/journal.pone.0135494)
Supplement: S2 Table — Genera listed were significantly different between the FORM and F+GP piglets. (DOCX) [file pone.0135494.s004.docx]

Table S2. Genera of ascending colon contents of 21d old piglets fed formula (FORM), formula supplemented with GOS and PDX (F+GP).^1^

|  | Diets | | | | | | |
| --- | --- | --- | --- | --- | --- | --- | --- |
|  | FORM | |  | F+GP | |  | ANOVA |
|  | Mean | SEM |  | Mean | SEM |  | p-value |
| *Catabacter* | 0.07 | 0.02 |  | 0.01 | 0.01 |  | < 0.01 |
| *Hydrogenanaerobacterium* | 0.97 | 0.23 |  | 0.25 | 0.11 |  | 0.02 |
| *Lactobacillus* | 0.06 | 0.02 |  | 0.71 | 0.44 |  | 0.04 |
| *Oscillospira* | 6.45 | 2.18 |  | 1.59 | 0.57 |  | 0.04 |
| *Parabacteroides* | 8.67 | 2.22 |  | 18.33 | 3.98 |  | 0.04 |
| *Ruminococcus* | 9.54 | 2.37 |  | 4.13 | 1.06 |  | 0.03 |

^1^ Genera included in analysis had a False Discovery Rate (FDR) < 0.1, data expressed as percent relative abundance
