# Supplementary material for: Characterization of the Intestinal Lactobacilli Community following Galactooligosaccharides and Polydextrose Supplementation in the Neonatal Piglet
Source: PLoS One. 2015 Aug 14;10(8):e0135494. doi: 10.1371/journal.pone.0135494 (PMC4537252; doi:10.1371/journal.pone.0135494)
Supplement: S3 Table — Values represent the relative abundance of Lactobacilli sp. (DOCX) [file pone.0135494.s005.docx]

Table S3. Lactobacilli detected in ileum and ascending colon of 21d old piglets fed formula (FORM), formula supplemented with GOS and PDX (F+GP) or sow-reared (SOW) as determined by 16S amplicon sequencing ^1,2^

|  | Ileum | | |  | Ascending Colon | | |
| --- | --- | --- | --- | --- | --- | --- | --- |
|  | FORM | F+GP | SOW |  | FORM | F+GP | SOW |
| *L. agilis* | 0.7 | 0.5 |  |  |  | 1.1 |  |
| *L. amylovorus* | 5.3 | 0.5 | 14.3 |  |  | 0.6 | 23.6 |
| *L. coleohominis*^3^ |  |  | 0.7 |  |  | 0.6 | 0.3 |
| *L. crispatus*^3^ |  |  |  |  |  | <0.1 |  |
| *L. curvatus*^3^ | 0.4 | 0.8 |  |  |  |  |  |
| *L. delbrueckii*^3^ | 1.0 | 0.9 | 0.8 |  |  |  | 1.0 |
| *L. equicursoris*^3^ | 0.5 | 0.1 | 0.8 |  |  |  |  |
| *L. fermentum*^3^ |  | 0.1 |  |  |  |  |  |
| *L. gasseri*^3^ |  | 0.6 |  |  |  |  |  |
| *L. johnsonii* | 19.2 | 31.8 | 29.8 |  | 60.9 | 22.5 | 36.3 |
| *L. mucosae* | 43.6 | 33.4 | 6.4 |  | 26.3 | 8.0 | 2.6 |
| *L. pentosus*^3^ | 0.4 |  |  |  |  |  |  |
| *L. plantarum* | 15.4 | 0.9 |  |  |  |  |  |
| *L. pontis*^3^ | 0.1 | 0.1 |  |  |  |  |  |
| *L. reuteri* | 1.6 | 7.6 | 17.0 |  |  | 1.0 | 3.1 |
| *L. rogosae* |  |  |  |  | 4.3 |  | 0.3 |
| *L. ruminis*^3^ |  |  | 0.3 |  |  |  |  |
| *L. salivarius*^3^ | 0.1 | 0.2 |  |  |  |  |  |
| *L. taiwanensis*^3^ |  |  |  |  |  | 0.3 |  |
| *L. vaginalis* | 7.3 | 20.4 | 1.0 |  | 5.9 | 0.3 |  |
| *Lactobacillus* sp. | 4.4 | 2.1 | 29.0 |  | 2.5 | 65.4 | 32.8 |

^1^ Data presented as percent relative abundance

^2^ Non-detected lactobacilli represented by blank cells

^3^ Aggregated as Minor *Lactobacillus* sp in Figure 4
